# Efficacy and safety of low-dose interleukin-2 in combination with methotrexate in patients with active rheumatoid arthritis: a randomized, double-blind, placebo-controlled phase 2 trial

**DOI:** 10.1038/s41392-022-00887-2

**Published:** 2022-03-07

**Authors:** Xiaoying Zhang, Miao Miao, Ruijun Zhang, Xu Liu, Xiaozhen Zhao, Miao Shao, Tian Liu, Yuebo Jin, Jiali Chen, Huixin Liu, Xia Zhang, Yun Li, Yunshan Zhou, Yue Yang, Ru Li, Haihong Yao, Yanying Liu, Chun Li, Yuhui Li, Limin Ren, Yin Su, Xiaolin Sun, Jing He, Zhanguo Li

**Affiliations:** 1 Department of Rheumatology and Immunology, Peking University People’s Hospital, 100044 Beijing, China; 2 Department of Rheumatology and Immunology, The First Affiliated Hospital of Wannan Medical College, 241000 Wuhu, Anhui China; 3 Department of Clinical Epidemiology and Biostatistics, Peking University People’s Hospital, 100044 Beijing, China; 4 Beijing Key Laboratory for Rheumatism Mechanism and Immune Diagnosis (BZ0135), Beijing, China; 5 Peking-Tsinghua Center for Life Sciences, Beijing, China; 6 State Key Laboratory of Natural and Biomimetic Drugs, School of Pharmaceutical Sciences, Peking University, Beijing, China

**Keywords:** Rheumatic diseases, Immunotherapy

## Abstract

Rheumatoid arthritis (RA) is an aggressive autoimmune arthritis, and current therapies remain unsatisfactory due to low remission rate and substantially adverse effects. Low-dose interleukin-2 (Ld-IL2) is potentially a therapeutic approach to further improve the disease. This randomized, double-blind, placebo-controlled trial was undertaken to evaluate the efficacy and safety of Ld-IL2 in patients with active RA. Patients were randomly assigned (1:1) to receive Ld-IL2, defined as a dose of 1 million IU, or placebo in a 12-week trial with a 12-week follow-up. Three cycles of Ld-IL2 or placebo were administered subcutaneously every other day for 2 weeks (a total of 7 doses), followed by a 2-week break. All patients received a stable dose of methotrexate (MTX). The primary outcomes were the proportion of patients achieving the ACR20, DAS28-ESR <2.6, and the change from baseline in CDAI or SDAI at week 24. Secondary endpoints included other clinical responses and safety. The primary outcomes were achieved in the per-protocol population. The improvements from baseline in CDAI and SDAI were significantly greater across time points for the Ld-IL2 + MTX group (*n* = 17) than for the placebo+MTX group (*n* = 23) (*P* = 0.018 and *P* = 0.015, respectively). More patients achieved ACR20 response in the Ld-IL2 + MTX group than those in the placebo+MTX group at week 12 (70.6% vs 43.5%) and at week 24 (76.5% vs 56.5%) (*P* = 0.014). In addition, low Treg and high IL-21 were associated with good responses to Ld-IL2. Ld-IL-2 treatment was well-tolerated in this study. These results suggested that Ld-IL2 was effective and safe in RA. ClinicalTrials.gov number: NCT 02467504.

## Introduction

Rheumatoid arthritis (RA) is a prevalent autoimmune disease characterized by progressive articular destruction and is notoriously difficult to treat with poor remission.^1,2^ Basic studies have shown that impaired immunological homeostasis plays a critical role in the development of RA.^3^ The distribution of regulatory T cells (Tregs) contributes to the disease activity of RA;^4^ effector T cells (Teff) are involved in the pathogenesis of RA, particularly impacting joint erosion.^5–7^ Clinically, methotrexate (MTX) is the first-line anchor drug for RA, but it is efficacious in only 19.8–25.4% of RA patients.^1^ It was also shown that 30–50% of patients need additional treatment.^8–10^ The ceiling phenomenon of low efficacy of MTX in RA is at least partially related to its interrupting effect on Tregs.^11^ A novel strategy to overcome the dilemma phenomenon of MTX is expected clinically.

The cytokine interleukin-2 (IL-2) is essential for the biogenesis and function of Tregs. The deficiency of Tregs can be promoted by low-dose interleukin-2 (Ld-IL2).^12,13^ A study showed that defects of Tregs from patients with RA were reversed by exogenous IL-2 in vitro.^14^ On the other hand, Ld-IL2 can inhibit Th17 cell proliferation, which is associated with the development of RA.^15^ Ld-IL2 treatment may be beneficial in RA.

We evaluated the potential effects and safety of Ld-IL2 along with MTX on RA in a randomized, double-blind, placebo-controlled trial for the first time. Furthermore, we identified factors in predicting potential responses to the treatment of Ld-IL2.

## Result

### Study population

**Fig. 1:**
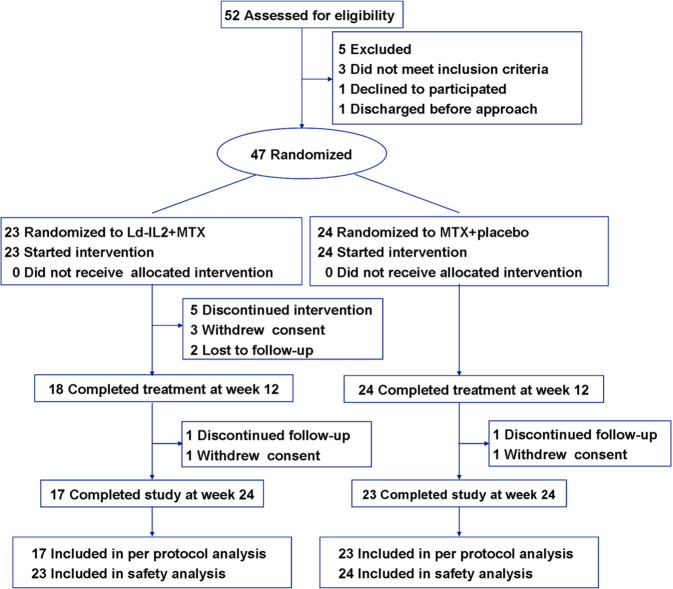
Consort flowchart of the study. Of the 47 randomized patients, 23 were exposed to Ld-IL2 (defined as a dose of 1 million IU) and 24 were exposed to placebo. All patients received methotrexate with Ld-IL2 or placebo. Ld-IL2 low-dose interleukin-2, MTX methotrexate

Of the 52 patients screened, 47 patients were randomly assigned to the Ld-IL2 + MTX group (*n* = 23) or the placebo+MTX (*n* = 24) (Supplementary Table 1). Flow diagram of participants at each stage of the trial was shown in Fig. 1. In total, 40 (85.1%) patients completed the 24-week trial. Through week 24, 7 patients discontinued the study; the reasons included withdrawal by patients (*n* = 5) and lost to follow-up (*n* = 2).

Baseline demographics and disease characteristics were comparable in the two groups (Table 1), with the following exception: compared with the placebo + MTX group, patients in the Ld-IL2 + MTX group have more swollen joint counts (4.5 versus 2.9).

**Table 1:** Baseline characteristics of patients with RA

	Intervention Arm^a^		
Characteristic	Ld-IL2 + MTX (*n* = 23)	Placebo+MTX (*n* = 24)	All participants (*N* = 47)
Female sex, *n* (%)	20 (87.0)	20 (83.3)	40 (85.1)
Age, years	52.6 ± 10.9	56.4 ± 9.9	54.5(10.5)
Height, m	1.60 (0.06)	1.64 (0.07)	1.62 (0.07)
Weight, kg	60.9 (9.5)	64.5 (9.7)	62.7 (0.9)
BMI, kg/m^2^	23.6 (2.7)	23.9 (2.8)	23.8 (2.7)
Duration of RA, years	11.7 (10.8)	9.3 (9.1)	10.5 (9.9)
DMARD naïve, *n* (%)	3 (13.0)	7 (29.2)	10 (21.3)
Pior DMARD therapy, *n* (%)			
LEF	12 (52.17)	13 (54.17)	25 (53.19)
SASP	6 (26.09)	3 (12.50)	9 (19.15)
HCQ	6 (26.09)	7 (29.17)	13 (27.66)
MTX initiated dose, mg/week	7.5	7.5	7.5
MTX maximum dose, mg/week	10.3 (1.37)	10.4 (1.41)	10.4 (1.38)
RF positive, *n* (%)	20 (87.0)	20 (83.3)	40 (85.1)
Anti-CCP antibody positive, *n* (%)	21 (91.3)	23 (95.8)	44 (93.6)
CRP, mg/L	21.60 (20.83)	15.32 (14.74)	18.39 (18.06)
ESR, mm/H	43.3 (25.9)	36.8 (24.9)	40.0 (25.3)
TJC (0–28)	8.9 (4.2)	8.8 (4.5)	8.9 (4.3)
SJC (0–28)^b^	4.5 (2.8)	2.9 (1.5)	3.7 (2.4)
Pain assessment, cm VAS	6.6 (2.0)	6.3 (2.4)	6.4 (2.2)
PhGA, cm VAS	6.5 (1.8)	6.0 (2.1)	6.2 (2.0)
PtGA, cm VAS	6.6 (1.8)	6.3 (2.3)	6.4 (2.1)
DAS28-ESR	5.55 (1.05)	5.26 (0.84)	5.40 (0.95)
CDAI score	26.7 (8.6)	24.0 (8.0)	25.3 (8.3)
SDAI score	28.83 (9.3)	25.53 (8.11)	27.15 (8.78)
HAQ-DI score	1.07 (0.66)	1.01 (0.65)	1.03 (0.65)
SF-36 PCS	23.06 (13.15)	25.14 (13.54)	24.12 (13.25)
SF-36 PMC	53.69 (11.18)	53.16 (13.55)	53.42 (12.31)

*BMI* body mass index (calculated as weight in kilograms divided by height in meters squared), *CCP* cyclic citrullinated peptide, *CDAI* Clinical Disease Activity Index, *CRP* C reactive protein, *DAS28* Disease Activity Score using 28 joints, *DMARD* disease-modifying anti-rheumatic drug, *ESR* erythrocyte sedimentation rate, *HAQ-DI* Health Assessment Questionnaire-Disability Index, *HCQ* hydroxycholoroquine, *Ld-IL2* low-dose interleukin-2, *LEF* leflunomide, *MTX* methotrexate, *PtGA* patient’s global assessment, *PhGA* physician’s global assessment, *RA* rheumatoid arthritis, *RF* rheumatoid factor, *SASP* salazosulfapyriding, *SDAI* Simplified Disease Activity Index, *SF-36 PCS* Short Form-36 physical component scores, *SF-36 MCS* Short Form-36 mental component scores, *SJC* swollen joint count, *TJC* tender joint count, *VAS* Visual Analogue Scale

^a^Data are presented as Mean (SD) unless stated otherwise

^b^*P* = 0.018, No other statistically significant differences were observed among two group

### Efficacy

**Fig. 2:**
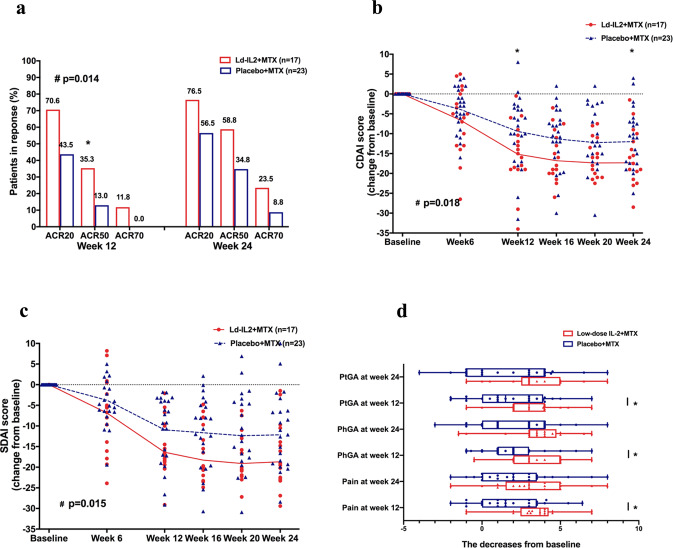
Clinical responses to Ld-IL2 combined with MTX therapy. The data were presented in a per-protocol (PP) analysis set. The proportion of patients achieving an ACR20/50/70 response by at week 12 and 24 (**a**). The mean changes from baseline for CDAI and SDAI (**b, c**). The mean changes from baseline for pain, PtGA and PhGA of disease activity (**d**). Data in graphs were mean ± SE. *P < 0.05, with analyses with a logistic regression model or a covariance (ANCOVA) model at week 12 or 24. ^#^*P*-value, presenting treatment differences across time points with a mixed model for repeated-measures analysis or Generalized Estimation Equations (GEE) method. ACR20/50/70 the American College of Rheumatology for 20%/50%/70% improvement, CDAI Clinical Disease Activity Index, SDAI Simplified Disease Activity Index, Ld-IL2 low-dose interleukin-2, MTX methotrexate, PtGA patient’s global assessment, PhGA physician’s global assessment

The primary outcomes were achieved in a per-protocol (PP) analysis set. More patients achieved American College of Rheumatology (ACR)20 response in the Ld-IL2 + MTX group (*n* = 17) than those in the MTX + placebo group (*n* = 23) at week 12 (70.6% vs 43.5%, *P* = 0.081) and at week 24 (76.5% vs 56.5%, *P* = 0.080). There were significant treatment differences across time points (*P* = 0.014). At week 12, the rate of ACR50 response in the Ld-IL2 + MTX group was significantly higher than that in the MTX + placebo group (*P* = 0.026). Compared to the placebo + MTX group, a higher percentage of patients with Ld-IL2 + MTX achieved ACR50 response (58.8% vs 34.8%) and ACR70 response (23.5% vs 8.8%) at week 24 (Fig. 2a, Supplementary Table 2), even though there were no statistically significant differences. The improvements from baseline in Clinical Disease Activity Index (CDAI) and Simplified Disease Activity Index (SDAI) were significantly greater across time points for the Ld-IL2 + MTX group than for the placebo+MTX group (*P* = 0.018 and *P* = 0.015, respectively, Fig. 2b, c, Supplementary Table 2). The change from baseline in CDAI score at week 24 was −16.6 in the Ld-IL2 + MTX group, as compared with −12.5 in the placebo+MTX group (*P* = 0.047). The changes from baseline in SDAI score at week 24 were −17.5 and −13.0 in the two groups, respectively (*P* = 0.062). At week 24, disease activity score (28 joint) calculated using erythrocyte sedimentation rate formula (DAS28-ESR) < 2.6 was achieved by 35.3% (6/17) of the patients in the Ld-IL2 + MTX group and 26.1% (6/23) of patients in the placebo+MTX group. Even though there was a higher percentage of patients achieving DAS28 remission in the Ld-IL2 group, statistically significant improvement was not observed.

Compared with the placebo group, benefits with Ld-IL2 were observed in pain assessment, physician’s and patient’s global assessment of disease activity at week 12 (*P* = 0.009, *P* = 0.033, and *P* = 0.006, respectively, Fig. 2d, Supplementary Table 2). No significant differences were found when comparing the decreases of tender joint count (TJC) and swollen joint count (SJC) between the two groups. Similar clinical improvements in acute phase reactants, Short Form-36 (SF-36) score, and health assessment questionnaire disability index (HAQ-DI) were shown for both Ld-IL2 and placebo groups. No significant decrease in rheumatoid factor (RF) and anti-cyclic citrullinated peptide (CCP) antibody was observed (Supplementary Table 2).

Intention-to-treat (ITT) analyses for clinical outcomes were also performed (Supplementary Table 3). Although some numerical trends towards improvement were observed in primary and secondary outcomes, the marginal changes were relatively small.

### Safety

Ld-IL2 was well-tolerated during the trial. Through week 24, the proportion of patients with at least one adverse event (AE) in the Ld-IL2 group was 47.8% (11/23) and in the placebo group was 37.5% (9/24) (Table 2). No drug-related serious adverse events (SAEs) occurred in both groups. Transient fever was observed in the Ld-IL2 + MTX group (*n* = 2), and there were injection-site reactions (3 in Ld-IL2 + MTX and 2 in placebo + MTX groups, respectively). No intervention was needed to resolve these events.

**Table 2:** Adverse events in the safety population^a^

Adverse events	Ld-IL2 + MTX (*n* = 23)	Placebo + MTX (*n* = 24)
All events, no. (%)		
≥1 Adverse event	11 (47.8)	9 (37.5)
≥1 Serious adverse event	2 (8.7)	1 (4.2)
Adverse event leading to discontinuation	2 (8.7)	0
Adverse event type, no. (%)		
Injection-site reactions	3 (13.0)	2 (8.3)
Fever after injection	2 (8.7)	0
Gastrointestinal disorders	2 (8.7)	1 (4.2)
Upper respiratory tract infection	1 (4.3)	1 (4.2)
Hepatic enzyme increased^b^	1 (4.3)	1(4.2)
Worsening of RA	1 (4.3)	3 (12.5)

*Ld-IL2* low-dose interleukin-2, *MTX* methotrexate, *RA* rheumatoid arthritis

^a^Adverse events were collected at each visit via inquiry and clinical laboratory tests. The safety population included all patients who were randomized and received at least 1 dose of study drug. If a patient had multiple types of adverse events, he/she was counted once for each type

^b^Aspartate aminotransferase or alanine aminotransferase at least three times the upper limit of normal

The incidence of patients with SAEs through week 24 was low and similar between the two groups. There were two SAEs reported in the Ld-IL2 group, including surgery for osteochondroma not related to the study and worsening of RA. The patient with worsening of RA had severe disease activity (DAS28-ESR 6.6) at baseline and was admitted to the hospital 4 weeks after the participation in the study. One patient with SAE in the placebo group was admitted to the hospital because of hypertension. All SAEs were singular events, and no specific association between SAEs and treatment was identified.

### Factors predicting potential response to Ld-IL2

**Fig. 3:**
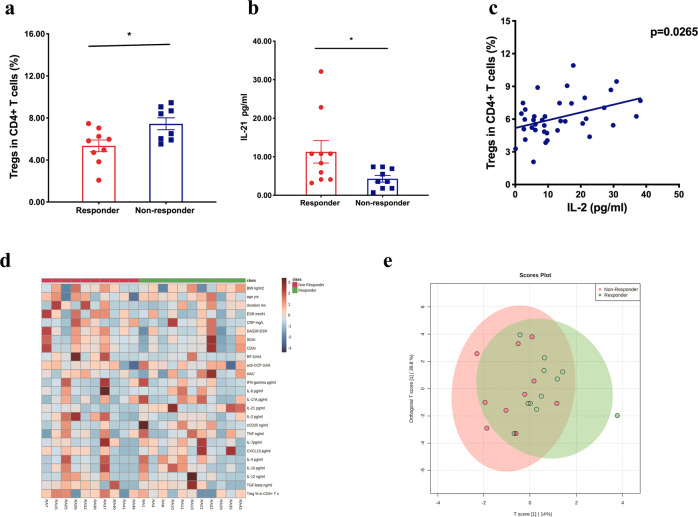
Predictive biomarkers for potential response to Ld-IL2 treatment in RA. The percentage of regulatory T cells (Tregs) in CD4+ T cells and serum level of IL-21 in responders and non-responders (**a, b**). The correlation between percentage of Tregs and serum level of IL-2 (**c**). Heatmap showed a classification of the two groups (**d**). Each column represented an individual. Colors in the horizontal bar denoted the non-responder group (red) and the responder group (green). Tiles were colored based on clinical features, Tregs and serum cytokine levels, red and blue indicating high and low levels, respectively. Primary composition analysis (**e**). The non-responder group was shown in red, and the responder group was shown in green. PC1 and PC2 account for 39.8% and 14%, respectively, of the total variance. Panels **d** and **e** were performed by R 4.1.0 and R-packages (mixOmics and pheatmap) (http://www.metaboanalyst.ca). Data in graphs were mean ± SE. *P < 0.05

To identify factors predicting potential response to Ld-IL2 in RA, we separated Ld-IL2 + MTX group (*n* = 23) into responders (*n* = 11) and non-responders (*n* = 10), according to the decrease of DAS28-ESR more than 1.2. Two patients who discontinued prior to receiving 1 cycle of Ld-IL2 were not involved. Improved response to the Ld-IL2 therapy was clearly correlated with a lower frequency of baseline Tregs and higher serum levels of IL-21 (*P* = 0.036 and *P* = 0.012, respectively, Fig. 3a, b, Supplementary Table 4). The percentage of Tregs was associated with the level of IL-2 (Fig. 3c). The detailed differences between the two groups were shown in Fig. 3d, e.

### Immunological analysis

**Fig. 4:**
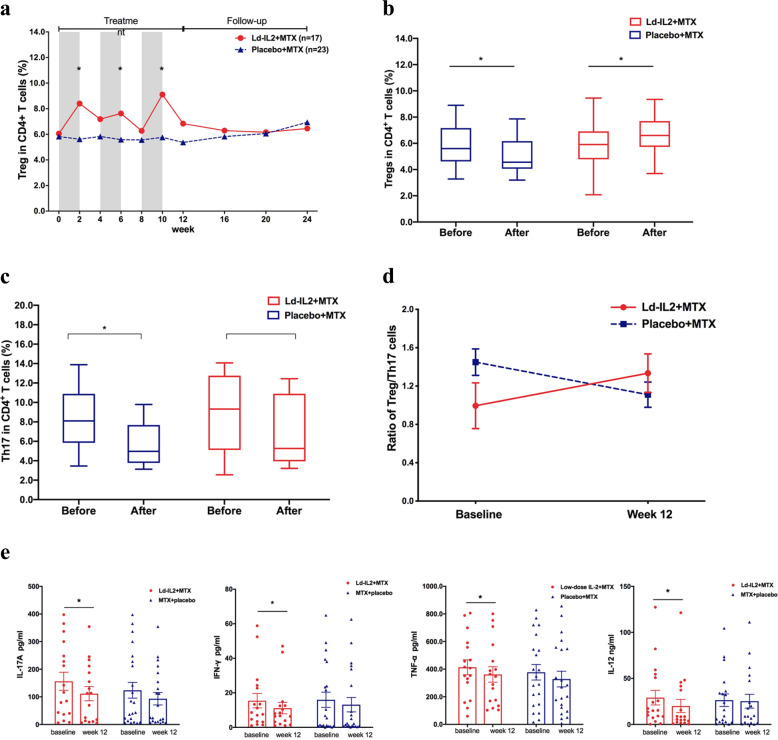
Ld-IL2 therapy synergized with MTX to expand the population of Tregs and ameliorate inflammation of RA. The proportion of Tregs in CD4+ T cells. Grey areas indicated the periods on Ld-IL2 or placebo therapy (**a**). The changes of the proportion of Tregs or Th17 in Ld-IL2 combined with MTX group and MTX alone group (**b, c**). The ratio of Tregs/Th17 (**d**). The serum levels of IL-17A, IFN-γ, TNF-α, and IL-12 were decreased significantly at week 12 (**e**). Data in bar graphs were mean ± SE. Ld-IL2 low-dose interleukin-2, MTX methotrexate, Treg regulatory T cell. *P < 0.05

We evaluated the changes of Treg cells in patients during the trial. Consistent with clinical improvement, the proportion of Treg cells in CD4^+^ T cells were significantly increased in association with the Ld-IL2 administration, at week 2, 6, and 10 (*P* = 0.001, 0.019, and 0.001, respectively, Fig. 4a). It was shown that MTX caused a significant decrease in Tregs (*P* = 0.029), while the addition of Ld-IL2 reversely expanded Tregs (*P* = 0.034) (Fig. 4b). The proportion of Th17 cells decreased significantly with MTX therapy (*P* = 0.001, Fig. 4c). There was a decline of Th17 cells in the Ld-IL2 + MTX arm, but it was not statistically significant (*P* = 0.078, Fig. 4c). The ratio of Treg/Th17 increased signifsignificantly with Ld-IL2 + MTX (*P* = 0.016), while it decreased with placebo+MTX (*P* < 0.001) (Fig. 4d). These data were consistent with the notion that Ld-IL2 counteracted the impairment of Tregs caused by MTX.

Notably, we also found that the therapy comprising Ld-IL2 and MTX caused significant reductions of inflammatory cytokines, including IL-17A, IFN-γ, TNF-α, and IL-12 levels, at week 12 (*P* = 0.004, 0.028, 0.020, and 0.015, respectively, Fig. 4e, Supplementary Table 5).

## Discussion

This randomized, double-blind, placebo-controlled clinical study provided the first evidence that Ld-IL2 further increased the efficacy of MTX. The treatment was well-tolerated, and was accompanied by a significant clinical improvement of CDAI, SDAI, and ACR response.

Tregs could be an ideal target for therapies to induce remission of autoimmune disorders.^16^ IL-2 has been reported to exert beneficial effects for the treatment.^12,17–19^ Recently, Klatzmann *et al*. reported the safety, biological and clinical effects of Ld-IL2 in a basket of 11 individual diseases, including RA.^20^ In the present study, we conducted a randomized, double-blind, placebo-controlled study. The results demonstrated that Ld-IL2 in combination with MTX outperformed the placebo+MTX for clinical outcomes including CDAI, SDAI, and ACR response.

The improvements of patient’s assessment of pain, patient’s global assessment (PtGA), and physician’s global assessment (PhGA) of disease activity were observed in the Ld-IL2 group, without significant reductions in TJC and SJC compared to the placebo group. TJC and SJC were important because articular involvement was the main feature of RA. However, systemic clinical manifestations, like fatigue and morning stiffness, were not involved. SDAI and CDAI incorporate the systemic features and are able to express the response of the study drug objectively. Our study demonstrated that Ld-IL2 treatment achieved a significant clinical improvement in response to SDAI, CDAI, and ACR response.

In agreement with improvements in clinical features, inflammatory cytokines were dampened with Ld-IL2 treatment. The dose and schedule of IL-2 administration varied in different studies and further clinical trials would be required to decipher the dose-response relationship.^21^ We defined three cycles of Ld-IL2 administration subcutaneously every other day for 2 weeks (a dose of 1 million IU, a total of 7 doses), followed by a 2-week break. We found an increase in the number of Tregs at the end of the treatment cycle, followed by a decrease until the next cycle. This treatment regimen was effective and tolerate in systemic lupus erythematosus (SLE).^22^

Given that the proportion of Tregs was known to be reduced in the peripheral blood of RA patients,^23^ promoting the Treg population could facilitate the development of novel immunotherapies for treating RA. The results of this study supported that MTX actually caused a significant decrease in Tregs, while administration of Ld-IL2 with MTX increased the percentage of Tregs. Data of in vitro assays supported that MTX decreased the proportion of Tregs, while administration of IL-2 with MTX increased Tregs. Furthermore, the level of Foxp3 and IL-10 mRNA expression decreased with MTX, and increased with IL-2, consistently with the change of Treg cell population (Supplementary Fig. 1). We assumed that the Treg-disrupting effect by MTX was at least partially the explanation for MTX resistance and the poor responding in patients with RA. There were inconsistent results of MTX effect on Tregs population and function.^24–26^ Many factors, such as background therapy, duration of MTX treatment, disease activity, and disease duration varied in previous studies, which could influence the results of MTX effect on the Treg frequency and function. As we know, one of the mechanisms of MTX efficacy is folate antagonism. Previous studies demonstrated that the Treg population was suppressed due to folate deficiency.^11,27^ We suggest that MTX impairs the Treg population by inhibiting the folate pathway. Further studies to define the mechanism of MTX to target Tregs are needed.

In addition, both MTX alone and Ld-IL2 + MTX treatment attenuated the percentage of Th17 cells. However, the ratio of Treg/Th17 significantly increased after the combination of Ld-IL2 and MTX treatment. The therapeutic effectiveness of MTX could be related to the decrease in circulating Th17 cell frequencies, while Ld-IL2 could counteract rebalance the immune homeostasis, suggesting a potential therapeutic strategy.

As a pleiotropic cytokine, high-dose IL-2, produced primarily by CD4 + T cells, can promote autoimmune responses, while low-dose IL-2 restores immune tolerance. Our study showed a decline in serum IL-2 level after Ld-IL2 treatment (Supplementary Table 5). This result suggested that Ld-IL2 might downregulate endogenous IL-2 and drive immune tolerance without increasing serum IL-2. A decrease in IL-2 concentration might result from the efficacy of the treatment, which needs to be further studied in a larger trial.

We observed that although Ld-IL2 synergized with MTX in improving the clinical and immunological outcomes of RA, in some patients Ld-IL2 failed to achieve adequate suppression of disease activity. We determined Ld-IL2 response in patients who had completed at least 1 cycle of Ld-IL2. In order to address this apparently differential response to the treatment, our results indicated that response to Ld-IL2 was probably correlated with a lower proportion of Treg cells and higher serum IL-21 levels. IL-21 plays a critical role in the activation and proliferation of Th17 cells and follicular helper T (Tfh) cells and further mediates several inflammatory processes in RA pathogenesis and progression.^28^ IL-21 could inhibit IL-2 production and impair Treg homeostasis,^29^ while IL-2 could improve these effects. Thus, optimizing the stratification will help to understand the conditions in which patients can benefit from Ld-IL2 therapy.

Current treatment regimens mainly rely on conventional synthetic disease-modifying anti-rheumatic drugs (csDMARDs) and biologic agents, which are associated with substantial adverse effects including liver damage, cytopenia, nausea, and various infections.^1,30^ MTX is the most widely accepted initial therapy, but it is not efficacious in a large proportion of patients. The potential adverse effects limit the use of MTX. In this study, gastrointestinal disorders and hepatic enzyme increase were associated with the side effects of MTX. A drug-related side effect was barely observed in Ld-IL2, which was consisted with previous studies.^12,22^ Particularly, Ld-IL2 could be beneficial for leukopenia and infection. In SLE study, we observed the significant increase of natural killer (NK) cells in response to Ld-IL2 treatment, which implicated potential augmentation of anti-infection cellular immunity.^22^ We also found that CD56^bri^ NK cells were increased in association with Ld-IL2 administration (Supplementary Fig. 2). Injection-site reactions and transient fever were the most common AE, while no event was severe or resulted in the discontinuation of the study agent. Vascular leak syndrome, one of the main complications of high-dose IL-2 administration, did not occur. Overall, no safety risk or a particular pattern of association between AEs and Ld-IL2 treatment was observed.

Six patients in the Ld-IL2 + MTX group and 1 patient in the placebo+MTX group discontinued the trial prematurely. The withdrawal might result from the protocol-mandated requirement for frequent visits. None of the patients reported premature discontinuation associated with the study drug.

Our study has some limitations. Enrollment was not stratified due to the small sample size in the study. In addition, the bias was possible because of the higher withdrawal rate in Ld-IL2 + MTX group. The background DMARD therapy varied among patients and was not further analyzed because of the inefficient number of patients. In a further study, optimizing the stratification would help to understand the clinical conditions that are likely to benefit from the Ld-IL2 therapy.

Taken together, this study provides the first clinical evidence that Ld-IL2 combined with MTX results in improvements in clinical and immunological responses of RA. The results demonstrate that Ld-IL2 is a novel and practical therapeutic approach in RA.

## Methods

### Study design

This study was a 6-month, pilot, randomized, double-blind, placebo-controlled trial to verify the clinical response and safety of Ld-IL2 for the treatment of moderate to severely active RA (ClinicalTrials.gov number, NCT02467504). This study was performed in accordance with the Declaration of Helsinki and Good Clinical Practice principles, and was approved by Peking University People’s Hospital Ethics Committee. All patients provided written informed consent. This study followed the Consolidated Standards of Reporting Trials (CONSORT) reporting guideline.^31^

### Participants

Methotrexate (MTX)-naïve patients with moderate to severely active RA were recruited from Department of Rheumatology and Immunology of Peking University People’s Hospital from July 2015 to August 2017. All patients (18–70 years) with RA fulfilled the revised 1987 ACR criteria,^32^ and were eligible if they had moderate to severely active disease, defined as DAS28-ESR > 3.2.^33^ Patients could continue treatments with oral non-steroidal anti-inflammatory drugs or oral corticosteroids (≤10 mg/day) and/or csDMARDs except MTX, provided that the doses had been stable for at least 3 months before baseline and remained stable throughout the trial. Patients previously given biologic DMARDs or MTX were excluded from the study. Patients were also excluded if they had an autoimmune disease other than RA, an active infection, recurrent bacterial infections, severe hepatic and renal dysfunction, or malignant tumor (Supplementary Table 6). Patients could withdraw from the study at any time for any reason.

### Randomization

Patients who underwent screening procedures and met the inclusion criteria were enrolled in the study. Stratified randomization was performed using computer-generated block randomization (block size of 4), by an independent third party (Beijing Stemexcel Technology Co. Ltd, China). Patients were randomly assigned (1:1) to Ld-IL2 + MTX or placebo+MTX. Patients, investigators, study coordinators, and monitors were masked to treatment assignment during subcutaneous administration of the drug and assessment of the patients during the 24-week trial.

### Interventions

Ld-IL2 (recombinant human IL-2Ser^125^) and placebo were sponsored by Beijing SL PHARM. None of the sponsors were involved in the design and conduction of this study, or the preparation of this manuscript. The data of this study would be unavailable to the sponsors until the study was published.

Ld-IL2 was administered as a dose of 1 million IU. Three cycles of Ld-IL2 or placebo were administered subcutaneously every other day for 2 weeks (a total of 7 doses), followed by a 2-week break. The dosing schedule was used in earlier studies^22,34^. MTX was initiated at 7.5 mg per week, and was increased to a maximum of 15 mg per week by week 4. MTX 7.5–15 mg per week was permitted for patients intolerant to higher doses. Patients received folic acid supplements of 10 mg per week. The study design was shown in Supplementary Fig. 3. Patients were assessed at a 2-week interval during the first 12 weeks and at a 4-week interval thereafter until week 24. Physical examination included SJC and TJC with 28 joints. The patient’s assessment of pain, PtGA, and PhGA of disease activity were evaluated using visual analog scales (VAS) of 0–10 cm. Physical function was assessed by HAQ-DI. Health-related quality of life, assessed at baseline and week 24, was evaluated using the SF-36, which assessed eight domains scored from 0 to 100 that could be aggregated into physical and mental component sores (PCS and MCS).^35^ Laboratory monitoring included measurement of inflammatory markers, blood counts, and routine biochemistry.

### Outcome measures

The primary outcomes were the proportion of patients achieving ACR20 response and DAS28 remission, the change from baseline in the CDAI and SDAI at week 24. Secondary outcomes included improvement of physical examination, the change of disease activity assessment using VAS, the change from baseline of DAS28-ESR, the change of physical function assessment by HAQ-DI score, and the improvement of quality of life using the SF-36.

ACR20/50/70 assessments were based on a 20%/50%/70% or greater improvement from baseline in the number of tender joints, a 20%/50%/70%, or more improvement in the number of swollen joints, and a 20%/50%/70% or greater improvement in 3 of the 5 remaining core set measures: patient’s assessment of pain, PtGA, PhGA, HAQ-DI, and CRP.

### Flow cytometry and ELISA assays

Peripheral blood mononuclear cells (PBMCs) were resuspended in Cell Staining Buffer at 5–10 × 10^6^ cells/ml and distributed 100 µl/tube of cell suspension (5–10 × 10^5^ cells/tube) into 12 × 75 mm plastic tubes. Cells were pre-incubated with 5 µl of Human TruStain FcX™ (BioLegend) per 100 µl of cell suspension for 5–10 min at room temperature. After washing one time, fluorescence conjugated antibodies were added at predetermined optimum concentrations and incubated on ice for 20 min in the dark. Cells were then washed two times with at least 2 ml of Cell Staining Buffer by centrifugation at 400 × *g* for 5 min. Cell pellets were resuspended in 0.5 ml of Cell Staining Buffer and 5 µl (0.25 µg)/million cells of 7-AAD solution was added to exclude dead cells. Incubate on ice for 3–5 min in the dark. Proportions of T cell subsets were analyzed by flow cytometry using a FACSAria II (BD) instrument and FlowJo software (TreeStar). The fluorescence conjugated antibodies for flow cytometry were listed in Supplementary Table 7. Tregs were defined as CD3^+^CD4^+^CD25^high^CD127^low^ and Th17 cells were defined as CD3^+^CD4^+^CD45RA^-^CXCR3^-^CCR6^+^ (Supplementary Fig. 4).^36,37^ Inflammation associated cytokines or other soluble factors in patient serum, including IL-2, IL-6, IL-17A, IL-21 TNF-α, IFN-γ, sCD25, IL-4, IL-12, TGF-β, and CXCL13, were determined by ELISA kits (MultiSciences, Hangzhou, China), following instructions from the manufacturer.

### Adverse events

Safety was evaluated by the frequency and severity of AEs through 24 weeks. Patients were monitored at each visit for vital signs, clinical and laboratory abnormalities, AEs, and SAEs.

### Statistical analysis

The sample size and power of the study were calculated based on the proportion of patients achieving ACR20 response in either group. Assuming that the proportion of patients receiving placebo + MTX group at week 24 would be 40% and the proportion of patients in Ld-IL2 + MTX group would be 75%, 22 patients were required for each group to have 70% or higher power to detect the difference between the two groups, with a significance level of 0.05.

Analysis of efficacy was performed on PP and ITT populations. The PP population was defined as the patients who completed the entire 24-week trial. The ITT population consisted of patients who were randomized and took at least one dose of treatment. If a patient had data for at least one ACR component at week 24, missing component data were imputed with the last observation carried forward if baseline data were available; otherwise, missing components were considered to worse than ACR20 response. The safety population included all patients who received at least one Ld-IL2 or placebo infusion.

Statistical analyses for baseline demographic and disease characteristics were done using student *t*-tests, Mann-Whitney U for comparisons of continuous variables, and Chi-square test for comparison of categorical variables. Binary efficacy variables were analyzed with a logistic regression model, adjusting age, gender, and baseline DAS28-ESR score. Continuous variables were assessed with an analysis of covariance (ANCOVA) model, including treatment group and baseline score. For continuous variables, treatment differences across time points were evaluated using a mixed model for repeated-measures analysis, with a visit, treatment group, treatment-by-visit interactions included in the model. The generalized Estimation Equations (GEE) method in a logistic repeated-measures model was used for categorical variables, controlling for confounder variables. Principal component analysis (PCA) and cluster analysis were used to analyze the variance between response and non-response groups to Ld-IL2 + MTX treatment. Cluster analysis by heatmap and PCA was performed by R 4.1.0 and R-packages (mixOmics and pheatmap) (http://www.metaboanalyst.ca).

A nominal significance level of 0.05 (two-sided) was applied to all the analyses. All statistical analyses were carried out by SPSS (version 23, IBM) and Graph Pad Prism (Version 5.0, Graph Pad Software).

## Supplementary information

supplemental material

## Data Availability

The data supporting the findings in this study will be available from the corresponding author on request.
