# Supplementary material for: Efficacy and safety of low-dose interleukin-2 in combination with methotrexate in patients with active rheumatoid arthritis: a randomized, double-blind, placebo-controlled phase 2 trial
Source: Signal Transduct Target Ther. 2022 Mar 7;7:67. doi: 10.1038/s41392-022-00887-2 (PMC8898945; doi:10.1038/s41392-022-00887-2)
Supplement: Supplementary file 1 — supplemental material [file 41392_2022_887_MOESM1_ESM.docx]

Supplementary Materials for Efficacy and safety of low-dose interleukin-2 in combination with methotrexate in patients with active rheumatoid arthritis: a randomized, double-blind, placebo-controlled phase 2 trial

Xiaoying Zhang, Miao Miao, Ruijun Zhang, Xu Liu, Xiaozhen Zhao, Miao Shao, Tian Liu, Yuebo Jin, Jiali Chen, Huixin Liu, Xia Zhang, Yun Li, Yunshan Zhou, Yue Yang, Ru Li, Haihong Yao, Yanying Liu, Chun Li, Yuhui Li, Limin Ren, Yin Su, Xiaolin Sun, Jing He,^△^ Zhanguo Li^△^

^△^Correspondence to:

Professor Jing He, Email: hejing1105@126.com.

Professor Zhanguo Li, Email: li99@bjmu.edu.cn.

**This PDF file includes:**

[Fig. S1. Ld-IL2 expanded the population of Tregs. 2](#_Toc92266779)

[Fig. S2. Ld-IL2 therapy increased the proportion of CD56^bri^NK cells. 3](#_Toc92266780)

[Fig. S3. Study design. 4](#_Toc92266781)

[Fig. S4. Representative gating of Tregs and Th17 cells. 5](#_Toc92266782)

[Table S1. Clinical Characteristics of the Patients with RA in the Study. 6](#_Toc92266783)

[Table S2. Primary and Secondary Per-protocol Outcomes Analysis.^a^ 9](#_Toc92266784)

[Table S3. Primary and Secondary Outcomes Analysis in ITT population.^a^ 12](#_Toc92266785)

[Table S4. Factors predicting potential response to low-dose interkleukin-2 treatment in RA. 15](#_Toc92266786)

[Table S5. Change in Cytokines after Low-dose IL-2 Treatment. 16](#_Toc92266787)

[Table S6. Inclusion and Exclusion Criteria. 18](#_Toc92266788)

[Table S7. Antibodies Used in Flow Cytometric Analysis. 20](#_Toc92266789)


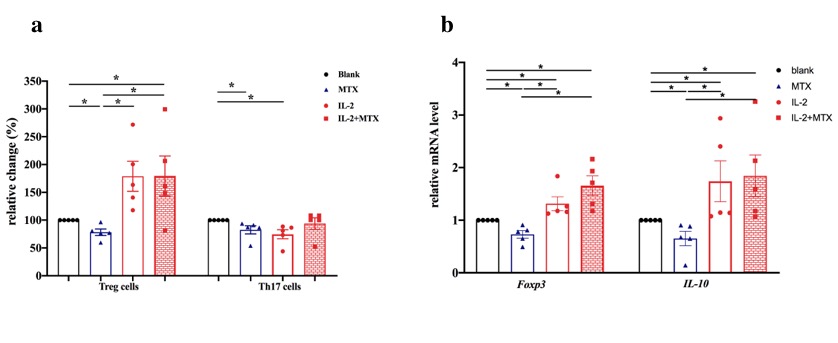


# Fig. S1. Ld-IL2 expanded the population of Tregs.

The relative change in Tregs and Th17 cells derived from PBMC of RA patients with MTX, IL-2, and IL-2+MTX, respectively (A). The percentage of Tregs or Th17 cells in blank was normalized to 100%. *Foxp3* and *IL-10* mRNA expression in PBMC (B).

Ld-IL2=low-dose interleukin-2; MTX=methotrexate; PBMC=peripheral blood mononuclear cell; Tregs= regulatory T cells.

**
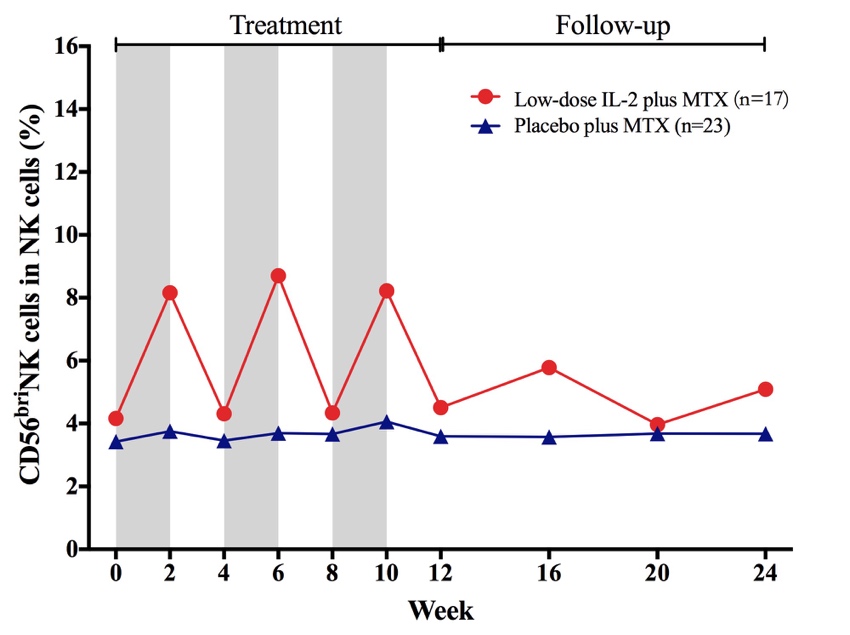
**

# Fig. S2. Ld-IL2 therapy increased the proportion of CD56^bri^NK cells.

Grey areas indicated the periods on Ld-IL2 or placebo therapy. The proportion of CD56^bri^NK cells (defined as CD56^bri^CD16^-^) in NK cells (defined as CD3^-^CD56^+^) was shown.

Ld-IL2=low-dose interleukin-2; MTX=methotrexate; NK=natural killer.


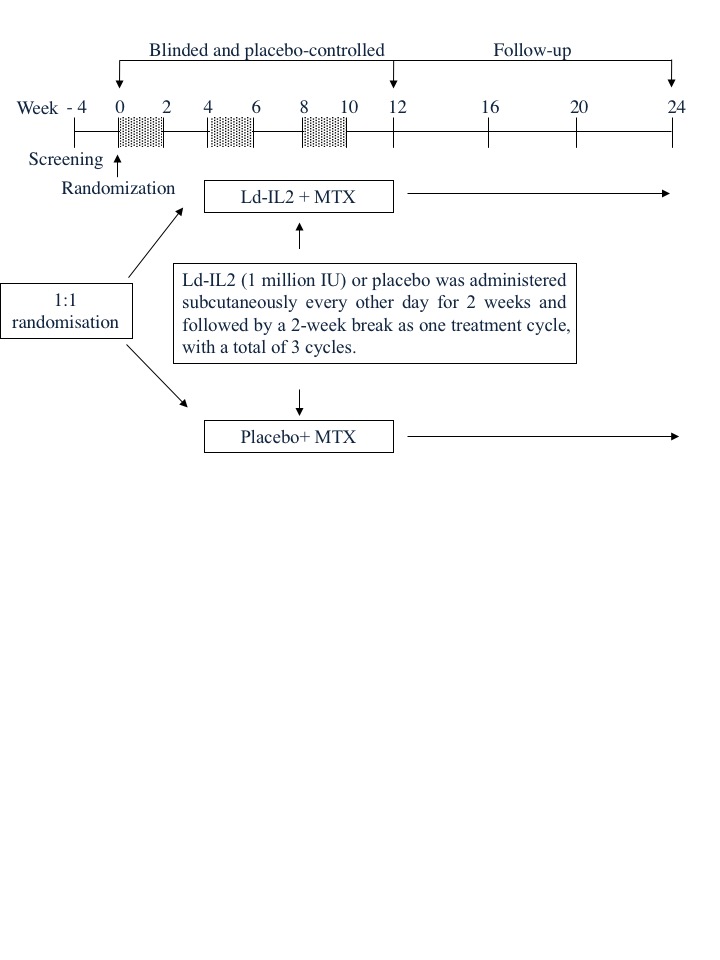


# Fig. S3. Study design.

A randomized, double-blind, placebo-controlled study was conducted. Three cycles of Ld-IL2 or placebo were administered subcutaneously every other day for 2 weeks (a total of 7 doses), followed by a 2-week break. Grey areas indicated the periods on Ld-IL2 or placebo therapy.

**
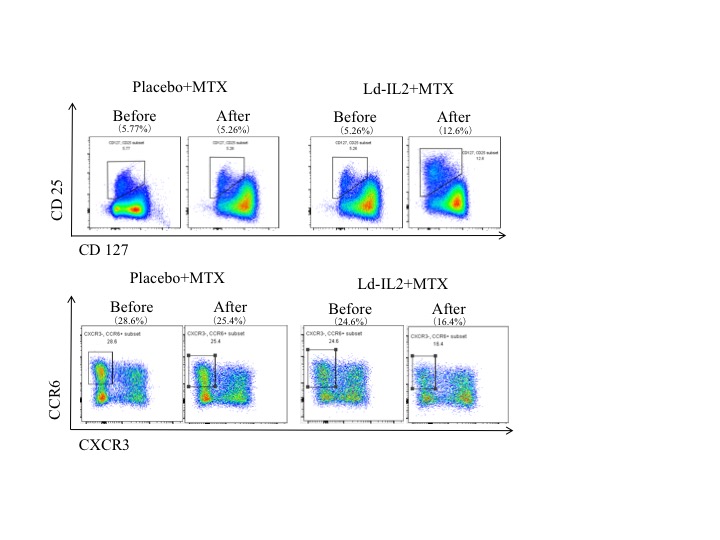
**

# Fig. S4. Representative gating of Tregs and Th17 cells.

The gating of Tregs and Th17 cells before and after treatment for patients.

# Table S1. Clinical Characteristics of the Patients with RA in the Study.

| **ID** | **Gender/Age (ys)** | | **Duration (months)** | **Extra-articular manifestation** | **Previous DMARDs, Glucocorticoids or NSAIDs treatment** | **MTX-maximum dose** | **Complication** | **Group** | **Enrollment date** | **AEs, SAEs or discontinued from study** |
| --- | --- | --- | --- | --- | --- | --- | --- | --- | --- | --- |
| 1 | F/57 | | 34 |  | Non | 10mg QW | Hypertension; Hyperlipidaemia | Ld-IL2 | 2015-10-22 |  |
| 2 | F/57 | | 7 |  | Non | 10mgQW |  | Placebo | 2015-11-5 |  |
| 3 | F/58 | | 5 |  | Non | 12.5mgQW |  | Ld-IL2 | 2016-1-29 | Injection site reaction |
| 4 | M/55 | | 32 |  | LEF 20mgQD | 12.5mgQW | Hypothyroidism | Placebo | 2016-4-20 |  |
| 5 | F/69 | | 7 |  | Non | 10mgQW | Rheumatic heart disease; Hyperuricemia | Placebo | 2016-4-25 |  |
| 6 | F/57 | | 151 |  | Prednisone 5mgQD | 10mgQW |  | Placebo | 2016-6-8 | Gastrointestinal disorder |
| 7 | F/58 | | 26 |  | LEF 20mgQD; HCQ 200mgBID; Prednisone 10mgQD | 10mgQW |  | Ld-IL2 | 2016-6-17 |  |
| 8 | F/52 | | 43 |  | TGP 600mgBID | 10mgQW |  | Ld-IL2 | 2016-7-15 | Injection site reaction |
| 9 | F/52 | | 32 |  | Non | 10mgQW |  | Placebo | 2016-8-19 |  |
| 10 | F/55 | | 94 |  | TWP 20mgTID | 10mgQW | Hypertension | Ld-IL2 | 2016-9-9 | Gastrointestinal disorder |
| 11 | F/45 | | 252 |  | Non | 12.5mgQW | Nephrectomy | Ld-IL2 | 2016-9-22 |  |
| 12 | M/38 | | 109 |  | LEF 20mgQD | 15mgQW |  | Placebo | 2016-12-12 | Worsening of RA |
| 13 | M/55 | | 13 | Rheumatoid nodule | TWP 20mgTID;  Loxoprofen sodium 60mgBID | 12.5mgQW |  | Ld-IL2 | 2016-12-13 |  |
| 14 | F/43 | | 133 |  | LEF 20mgQD; HCQ 200mgBID | 12.5mgQW |  | Placebo | 2016-12-15 | Upper respiratory tract infection |
| 15 | F/65 | | 450 |  | HCQ 200mgBID; Prednisone 5mgQD | 10mgQW |  | Ld-IL2 | 2016-12-17 | Fever after injection; |
| 16 | F/50 | | 5 |  | Non | 10mgQW |  | Placebo | 2016-12-22 | Injection site reaction |
| 17 | M/68 | | 25 | ILD | LEF 20mgQD; HCQ 200mgBID | 7.5mgQW | Hypertension; Diabetes mellitus | Placebo | 2016-12-23 | Hospitalized not related to RA |
| 18 | F/67 | | 65 |  | LEF 20mgQD; SSZ 500mgTID | 7.5mgQW |  | Ld-IL2 | 2016-12-27 | Withdrew consent |
| 19 | F/54 | | 231 |  | LEF 20mgQD | 10mgQW |  | Ld-IL2 | 2017-1-5 | Withdrew consent |
| 20 | F/64 | | 110 |  | LEF 20mgQD | 10mgQW |  | Placebo | 2017-1-10 |  |
| **Table S1. Continued.** | | | | | | | | | | |
| **ID** | **Gender/Age (ys)** | **Duration (months)** | | **Extra-articular manifestation** | **Previous DMARDs, Glucocorticoids or NSAIDs treatment** | **MTX-maximum dose** | **Complication** | **Group** | **Enrollment date** | **AEs, SAEs or discontinued from study** |
| 21 | F/59 | 183 | |  | LEF 10mgQD | 10mgQW |  | Ld-IL2 | 2017-1-12 | Hepatic enzyme increased |
| 22 | M/69 | 305 | | ILD | LEF 20mgQD; Prednisone 10mgQD | 10mgQW | Chronic gastritis | Ld-IL2 | 2017-1-12 |  |
| 23 | F/54 | 86 | |  | LEF 20mgQD; HCQ 200mgBID | 10mgQW |  | Placebo | 2017-1-16 |  |
| 24 | M/62 | 281 | |  | LEF 20mgQD; HCQ 200mgBID; SSZ 750mgBID | 10mgQW | Gastric ulcer | Placebo | 2017-1-16 |  |
| 25 | F/30 | 171 | |  | LEF 20mgBID; HCQ 200mgBID; SSZ 1gBID | 10mgQW |  | Ld-IL2 | 2017-1-20 | Hospitalized not related to RA; worsening of RA;  Withdrew consent |
| 26 | M/64 | 37 | | ILD; rheumatoid nodule | LEF 20mgBID; HCQ 200mgBIDl; Prednisone 5mgQD; Loxoprofen sodium 60mgBID | 7.5mgQW | Hypertension  Diabetes mellitus | Ld-IL2 | 2017-1-16 |  |
| 27 | F/59 | 62 | | ILD | HCQ 200mgBID | 10mgQW |  | Placebo | 2017-1-24 |  |
| 28 | F/63 | 75 | | ILD | LEF 10mgQD (1year ago) | 10mgQW |  | Placebo | 2017-2-5 | Worsening of RA |
| 29 | F/38 | 50 | |  | Non | 12.5mgQW |  | Ld-IL2 | 2017-2-4 |  |
| 30 | F/26 | 3 | |  | Non | 10mgQW |  | Placebo | 2017-1-24 | Injection site reaction; Withdrew consent |
| 31 | F/58 | 8 | |  | Non | 10mgQW |  | Placebo | 2017-2-5 |  |
| 32 | F/61 | 424 | |  | LEF 10mg QD; SSZ 750mgTID; Prednisone 5mgQD | 10mgQW | Hypertension | Ld-IL2 | 2017-2-9 | Injection site reaction |
| 33 | F/52 | 257 | |  | LEF 20mgQD; SSZ 750mgTID; TWP 20mgTID | 10mgQW | Hypertension | Placebo | 2017-2-5 | Worsening of RA |
| 34 | F/50 | 306 | |  | LEF 20mgQD | 10mgQW | Hypertension | Placebo | 2017-2-9 |  |
| 35 | F/54 | 147 | |  | HCQ 200mgBID | 10mgQW |  | Ld-IL2 | 2017-2-9 | Upper respiratory tract infection |
| 36 | F/41 | 75 | |  | SSZ 1g BID; TWP 20mg BID | 10mgQW |  | Ld-IL2 | 2017-2-16 | Lost to follow-up |
| 37 | F/52 | 282 | |  | LEF 20mgQD; SSZ 750mgTID | 10mgQW | Hypertension | Ld-IL2 | 2017-2-16 | Hospitalized not related to RA |
| 38 | F/64 | 257 | |  | LEF 20mgQD | 10mgQW | Hypertension | Placebo | 2017-2-23 |  |
| **Table S1. Continued.** | | | | | | | | | | |
| **ID** | **Gender/Age (ys)** | | **Duration (months)** | **Extra-articular manifestation** | **Previous DMARDs, Glucocorticoids or NSAIDs treatment** | **MTX-maximum dose** | **Complication** | **Group** | **Enrollment date** | **AEs, SAEs or discontinued from study** |
| 39 | F/65 | | 87 |  | LEF 10mgQD; SSZ 750mgTID; TGP 600mgTID; Loxoprofen sodium 60mgBID | 10mgQW | Gallstone | Placebo | 2017-2-23 |  |
| 40 | F/47 | | 50 |  | Loxoprofen sodium 60mgBID | 10mgQW |  | Ld-IL2 | 2017-2-20 | Withdrew consent |
| 41 | F/54 | | 51 |  | LEF 20mgQD | 10mgQW |  | Ld-IL2 | 2017-3-2 | Lost to follow-up |
| 42 | F/61 | | 391 |  | LEF 20mgQD | 10mgQW |  | Placebo | 2017-2-27 |  |
| 43 | F/47 | | 172 | Rheumatoid nodule | LEF 20mgQD; SSZ 1gBID | 10mgQW |  | Ld-IL2 | 2017-2-19 | Fever after injection; |
| 44 | M/69 | | 87 |  | HCQ 200mgBID | 10mgQW |  | Placebo | 2017-3-2 |  |
| 45 | F/60 | | 136 |  | HCQ 200mgBID; TWP 20mg BID | 12.5mgQW |  | Placebo | 2017-3-2 | Hepatic enzyme increased |
| 46 | F/27 | | 50 |  | HCQ 200mgBID; Prednisone 10mgQD | 12.5mgQW |  | Ld-IL2 | 2017-2-19 |  |
| 47 | F/46 | | 51 |  | Loxoprofen sodium 60mgBid | 10mgQW |  | Placebo | 2017-3-13 |  |

Abbreviations: AE, adverse event; DMARD, disease-modifying anti-rheumatoid drug; HCQ, hydroxycholoroquine; Ld-IL2, low dose interleukin-2; ILD, interstitial lung disease; LEF, Leflunomide; MTX, Methotrexate; NSAID, non-steroidal anti-inflammatory drug; RA, rheumatoid arthritis; SAE, severe adverse event; SSZ, Sulfasalazine; TGP, Total glucosides of paeony; TWP, Tripterygium wilfordii polyglycoside; ys, years.

# Table S2. Primary and Secondary Per-protocol Outcomes Analysis.^a^

|  | **Ld-IL2+MTX**  **(n=17)** | **Placebo+MTX**  **（n=23）** | **Difference (95%CI)** | **P-value** | **P-value^b^** |
| --- | --- | --- | --- | --- | --- |
| **ACR outcomes**, no. (%) |  |  |  |  |  |
| ACR20 |  |  |  |  |  |
| At week 12 | 12 (70.6) | 10 (43.5) | 0.288 (0.071 to 1.164) | 0.081 | **0.014** |
| At week 24 | 13 (76.5) | 13 (56.5) | 0.211 (0.037 to 1.202) | 0.080 |  |
| ACR50 |  |  |  |  |  |
| At week 12 | 6 (35.3) | 3 (13.0) | 0.077 (0.008 to 0.734) | **0.026** | 0.079 |
| At week 24 | 10 (58.8) | 8 (34.8) | 0.218 (0.047 to 1.014) | 0.052 |  |
| ACR70 |  |  |  |  |  |
| At week 12 | 2 (11.8) | 0 (0.0) | NA | 0.998 | 0.164 |
| At week 24 | 4 (23.5) | 2 (8.8) | 0.300 (0.042 to 2.135) | 0.229 |  |
| **CDAI score change from baseline** |  |  |  |  |  |
| At week 12 | -14.7 (-18.3 to -11.1) | -9.8 (-12.9 to -6.7) | 4.851 (0.085 to 9.616) | **0.046** | **0.018** |
| At week 24 | -16.6 (-19.7 to -13.6) | -12.5 (-15.1 to -9.9) | 4.097 (0.062 to 8.131) | **0.047** |  |
| **SDAI score change from baseline** |  |  |  |  |  |
| At week 12 | -15.5 (-18.8 to -12.3) | -11.6 (-14.4 to -8.9) | 3.965 (-0.364 to 8.293) | 0.071 | **0.015** |
| At week 24 | -17.5 (-21.0 to -13.6) | -13.0 (-16.1 to -10.0) | 4.433 (-0.229 to 9.095) | 0.062 |  |
| **DAS28-ESR endpoints**, no. (%) |  |  |  |  |  |
| DAS28-ESR<2.6 |  |  |  |  |  |
| At week 12 | 2 (11.8) | 1 (4.3) | 0.056 (0.001 to 2.824) | 0.149 | 0.462 |
| At week 24 | 6 (35.3) | 6 (26.1) | 0.368 (0.076 to 1.794) | 0.216 |  |
| DAS28-ESR≤3.2 |  |  |  |  |  |
| At week 12 | 7 (41.2) | 7 (30.4) | 0.521 (0.090 to 3.019) | 0.467 | 0.438 |
| At week 24 | 8 (47.1) | 9 (39.1) | 0.453 (0.104 to 1.967) | 0.291 |  |
| DAS28-ESR change from baseline |  |  |  |  |  |
| At week 12 | -1.69 (-2.13 to -1.24) | -1.27 (-1.65 to -0.89) | 0.416 (-0.174 to 1.006) | 0.162 | 0.337 |
| At week 24 | -2.09 (-2.60 to -1.57) | -1.78 (-2.22 to -1.34) | 0.303 (-0.372 to 0.978) | 0.369 |  |
| **Table S2. (Continued)** |  |  |  |  |  |
|  | **Ld-IL2+MTX**  **(n=17)** | **Placebo+MTX**  **（n=23）** | **Difference (95%CI)** | **P-value** | **P-value^b^** |
| **Percent change in ACR core components** |  |  |  |  |  |
| Tender joint count (0-28) |  |  |  |  |  |
| At week 12 | -63.3 (-79.5 to -47.1) | -47.0 (-60.9 to -33.0) | 0.163 (-0.051 to 0.377) | 0.131 | 0.385 |
| At week 24 | -77.1 (-91.3 to -62.9) | -66.2 (-78.4 to -54.0) | 0.109 (-0.077 to 0.296) | 0.243 |  |
| Swollen joint count (0-28) |  |  |  |  |  |
| At week 12 | -68.2 (-91.1 to -45.3) | -63.7 (-83.3 to -44.2) | 0.045 (-0.264 to 0.354) | 0.771 | 0.719 |
| At week 24 | -67.9 (-91.0 to -44.8) | -83.9 (-100.0 to -64.1) | 0.160 (-0.472 to 0.153) | 0.307 |  |
| Pain assessment, cm VAS |  |  |  |  |  |
| At week 12 | -51.3 (-68.5 to -34.0) | -20.4 (-35.2 to -5.5) | 0.309 (0.082 to 0.537) | **0.009** | 0.293 |
| At week 24 | -46.4 (-68.1 to -24.8) | -28.9 (-47.6 to -10.3) | 0.175 (-0.111 to 0.461) | 0.223 |  |
| PhGA, cm VAS |  |  |  |  |  |
| At week 12 | -50.5 (-64.8 to -36.3) | -30.0 (-42.2 to -17.8) | 0.205 (0.017 to 0.393) | **0.033** | **0.029** |
| At week 24 | -51.0 (-69.7to -32.2) | -31.6 (-47.7 to -15.5) | 0.194 (-0.054 to 0.441) | 0.121 |  |
| PtGA, cm VAS |  |  |  |  |  |
| At week 12 | -47.7 (-61.3 to -34.0) | -21.9 (-33.6 to -10.2) | 0.257 (0.077 to 0.437) | **0.006** | 0.095 |
| At week 24 | -51.3 (-84.6 to -18.1) | -9.3 (-37.9 to 19.3) | 0.420 (-0.019 to 0.859) | 0.060 |  |
| HAQ-DI |  |  |  |  |  |
| At week 12 | -31.7 (-59.2 to -4.2) | -16.1 (-39.8 to 7.5) | 0.156 (-0.207 to 0.519) | 0.390 | 0.090 |
| At week 12 | -60.1 (-108.7 to -11.6) | -9.2 (-50.9 to 32.6) | 0.510 (-0.131 to 1.150) | 0.116 |  |
| CRP |  |  |  |  |  |
| At week 12 | -34.5 (-78.2 to 9.2) | -29.8 (-64.7 to 5.2) | 0.047 (-52.4 to 61.9) | 0.867 | 0.142 |
| At week 24 | -31.3 (-83.1 to 20.5) | 3.3 (-41.0 to 47.7) | 0.346 (-0.344 to 1.036) | 0.316 |  |
| **SF-36 PCS change from baseline at week 24** | 16.44 (9.75 to 23.12) | 8.87 (3.13 to 14.62) | -7.565 (-16.39 to 1.26) | 0.091 |  |
| **SF-36 MCS change from baseline at week 24** | 3.87 (-1.46 to 9.20) | 4.68 (0.10 to 9.26) | 0.811 (-6.213 to 7.835) | 0.816 |  |
| **ESR change from baseline, mm/H** |  |  |  |  |  |
| At week 12 | -9.3 (-17.7 to -1.0) | -8.9 (-16.1 to -1.7) | 0.470 (-10.591 to 11.532) | 0.932 | 0.881 |
| At week 24 | -9.8 (-19.2 to -0.4) | -11.4 (-19.5 to -3.3) | -1.637 (-14.060 to 10.786) | 0.791 |  |
| **Table S2. (Continued)** |  |  |  |  |  |
|  | **Ld-IL2+MTX**  **(n=17)** | **Placebo+MTX**  **（n=23）** | **Difference (95%CI)** | **P-value** | **P-value^b^** |
| **RF change from baseline, IU/ml** |  |  |  |  |  |
| At week 12 | -154.2 (-842.0 to -20.1) | -35.0 (-182.5 to 24.6) | 620.6 (-48.81 to 1289.93) | 0.123 | 0.213 |
| At week 24 | -144.8 (-850.0 to -46.1) | -146.7 (-330.5 to 17.3) | 509.3 (-168.27 to 1186.89) | 0.432 |  |
| **IgA change from baseline at week 24, G/L** | -0.46 (-0.78 to -0.15) | -0.64 (-0.91 to -0.37) | -0.17 (-0.60 to 0.25) | 0.407 |  |
| **IgM change from baseline at week 24, G/L** | -0.12 (-0.29 to 0.04) | -0.17 (-0.31 to -0.03) | -0.05 (-0.26 to 0.17) | 0.678 |  |
| **IgG change from baseline at week 24, G/L** | -1.77 (-3.24 to -0.31) | -2.07 (-3.33 to -0.82) | -0.30 (-2.25 to 1.65) | 0.757 |  |
| **Anti-CCP antibody change from baseline at week 24, U/ml** | -14.3 (-32.7 to 4.1) | -35.1 (-50.9 to -19.3) | -20.830 (-45.141 to 3.481) | 0.090 |  |

^a^ Data are presented as least squares mean (95% CI) unless stated otherwise.

^b^ For continuous variables, treatment differences across time points were evaluated using a mixed model for repeated-measures analysis. For categorical variables, Generalized Estimation Equations (GEE) was used for analysis.

Abbreviations: ACR, American College of Rheumatology; EULAR, European League Against Rheumatism; ACR20/50/70, ≥20%/50%/70% improvement in the American College of Rheumatology criteria; CCP, cyclic citrullinated peptide; CDAI, Clinical Disease Activity Index; CI, confidence interval; CRP, C reactive protein; DAS28, Disease Activity Score using 28 joints; ESR, erythrocyte sedimentation rate; HAQ-DI, Health Assessment Questionnaire-Disability Index; Ig, immunoglobulin; Ld-IL2: low dose interleukin-2; MTX, methotrexate; PhGA, Physician’s global assessment of disease activity; PtGA, Patient’s global assessment of disease activity; RF, rheumatoid factor; SDAI, Simplified Disease Activity Index; SF-36 PCS, Short Form-36 physical component scores; SF-36 MCS, Short Form-36 mental component scores; SJC, swollen joint count; TJC, tender joint count; VAS, Visual Analogue Scale.

# Table S3. Primary and Secondary Outcomes Analysis in ITT population.^a^

|  | **Ld-IL2+MTX**  **(n=23)** | **Placebo+MTX**  **（n=24）** | **Difference (95%CI)** | **P-value** | **P-value^b^** |
| --- | --- | --- | --- | --- | --- |
| **ACR outcomes**, no. (%) |  |  |  |  |  |
| ACR20 |  |  |  |  |  |
| At week 12 | 12 (52.2) | 10 (41.7) | 2.076 (0.591 to 7.289) | 0.254 | 0.416 |
| At week 24 | 13 (56.5) | 13 (54.2) | 1.624 (0.435 to 6.066) | 0.471 |  |
| ACR50 |  |  |  |  |  |
| At week 12 | 6 (26.1) | 3 (12.5) | 12.720 (1.212 to 133.514) | **0.034** | 0.278 |
| At week 24 | 10 (43.5) | 8 (33.3) | 2.578 (0.637 to 10.439) | 0.184 |  |
| ACR70 |  |  |  |  |  |
| At week 12 | 2 (8.7) | 0 (0.0) | NA | 0.998 | 0222 |
| At week 24 | 4 (17.4) | 2 (8.3) | 3.020 (0.418 to 21.801) | 0.273 |  |
| **CDAI score change from baseline** |  |  |  |  |  |
| At week 12 | -10.5 (-14.2 to -6.7) | -9.8 (-13.5 to -6.1) | 0.670 (-4.627 to 5.968) | 0.800 | 0.834 |
| At week 24 | -11.6 (-15.4 to -7.8) | -12.5 (-16.2 to -8.8) | -0.879 (-6.234 to 4.475) | 0.742 |  |
| **SDAI score change from baseline** |  |  |  |  |  |
| At week 12 | -10.8 (-14.6 to -7.0) | -11.5 (-15.2 to -7.8) | -0.718 (-6.066 to 4.629) | 0.788 | 0.743 |
| At week 24 | -11.9 (-16.2 to -7.6) | -13.0 (-17.2 to -8.8) | -1.133 (-7.186 to 4.920) | 0.708 |  |
| **DAS28-ESR endpoints**, no. (%) |  |  |  |  |  |
| DAS28-ESR<2.6 |  |  |  |  |  |
| At week 12 | 2 (8.7) | 1 (4.2) | 19.561 (0.357 to 1070.407) | 0.145 | 0.462 |
| At week 24 | 6 (26.1) | 6 (25.0) | 2.295 (0.467 to 11.267) | 0.306 |  |
| DAS28-ESR≤3.2 |  |  |  |  |  |
| At week 12 | 7 (30.4) | 7 (29.2) | 1.794 (0.362 to 8.889) | 0.474 | 0.941 |
| At week 24 | 8 (34.8) | 9 (37.5) | 1.733 (0.414 to 7.246) | 0.451 |  |
| DAS28-ESR change from baseline |  |  |  |  |  |
| At week 12 | -1.17 (-1.62 to -0.72) | -1.26 (-1.70 to -0.82) | -0.087 (-0.722 to 0.547) | 0.783 | 0.699 |
| At week 24 | -1.42 (-1.97 to -0.87) | -1.77 (-2.31 to -1.23) | -0.354 (-1.127 to 0.419) | 0.361 |  |
| **Table S3. (Continued)** |  |  |  |  |  |
|  | **Ld-IL2+MTX**  **(n=23)** | **Placebo+MTX**  **（n=24）** | **Difference (95%CI)** | **P-value** | **P-value^b^** |
| **Percent change in ACR core components** |  |  |  |  |  |
| Tender joint count (0-28) |  |  |  |  |  |
| At week 12 | -36.7 (-59.3 to -14.1) | -47.4 (-69.5 to -25.3) | -10.7 (-42.3 to 20.9) | 0.497 | 0.280 |
| At week 24 | -45.5 (-68.7 to -22.2) | -65.8 (-88.6 to -43.1) | -20.4 (-52.9 to 12.2) | 0.213 |  |
| Swollen joint count (0-28) |  |  |  |  |  |
| At week 12 | -47.4 (-70.7 to -24.1) | -61.6 (-84.5 to -38.8) | -14.2 (-48.0 to 19.5) | 0.400 | 0.216 |
| At week 24 | -48.9 (-73.2 to -24.6) | -80.3 (-104.0 to -56.5) | -31.4 (-66.6 to 3.8) | 0.079 |  |
| Pain assessment, cm VAS |  |  |  |  |  |
| At week 12 | -39.1 (-54.5 to -23.6) | -20.5 (-35.6 to -5.3) | 18.6 (-3.0 to 40.3) | 0.090 | 0.702 |
| At week 24 | -33.2 (-51.8 to -14.5) | -28.9 (-47.1 to -10.6) | 4.3 (-21.9 to 30.5) | 0.742 |  |
| Physician’s GA, cm VAS |  |  |  |  |  |
| At week 12 | --35.6 (-49.9 to 21.3) | -28.0 (-42.0 to -14.0) | 7.6 (-12.5 to 27.7) | 0.448 | 0.286 |
| At week 24 | -34.0 (-51.4 to -16.5) | -32.0 (-49.1 to -14.9) | 2.0 (-22.5 to 26.5) | 0.870 |  |
| Patient’s GA, cm VAS |  |  |  |  |  |
| At week 12 | -35.8 (-48.4 to -23.2) | -22.9 (-35.2 to -10.6) | 12.9 (-4.7 to 30.6) | 0.147 | 0.469 |
| At week 24 | -35.5 (-63.4 to -7.6) | -11.3 (-38.5 to 16.0) | 24.2 (-14.8 to 63.3) | 0.218 |  |
| HAQ-DI |  |  |  |  |  |
| At week 12 | -27.2 (-592.2 to -2.2) | -8.1 (-32.5 to 16.4) | 19.1 (-16.9 to 55.2) | 0.291 | 0.138 |
| At week 12 | -50.1 (-90.9 to -9.4) | 2.1 (-37.8 to 42.0) | 52.2 (-6.6 to 111.1) | 0.080 |  |
| CRP, mg/L |  |  |  |  |  |
| At week 12 | 27.7 (-24.1 to 79.6) | -35.3 (-83.8 to 13.1) | -63.1 (-134.9 to 8.8) | 0.084 | 0.691 |
| At week 24 | 39.2 (-27.9 to 106.3) | -3.2 (-68.8 to 62.5) | -42.3 (-137.0 to 52.3) | 0.372 |  |
| **SF-36 PCS change from baseline at week 24** | 11.57 (5.69 to 17.44) | 9.29 (3.54 to 15.04) | -2.28 (-10.51 to 5.96) | 0.580 |  |
| **SF-36 MCS change from baseline at week 24** | 2.59 (-1.74 to 6.91) | 3.63 (-0.60 to 7.86) | 1.05 (-5.00 to 7.09) | 0.729 |  |
| **ESR change from baseline, mm/H** |  |  |  |  |  |
| At week 12 | -3.9 (-11.3 to 3.6) | -9.4 (-16.6 to -2.1) | -5.45 (-15.92 to 5.01) | 0.299 | 0.473 |
| At week 24 | -3.9 (-12.3 to 4.6) | -12.1 (-20.4 to -3.8) | -8.25 (-20.14 to 3.63) | 0.169 |  |
| **Table S3. (Continued)** |  |  |  |  |  |
|  | **Ld-IL2+MTX**  **(n=23)** | **Placebo+MTX**  **（n=24）** | **Difference (95%CI)** | **P-value** | **P-value^b^** |
| **RF change from baseline, IU/ml** |  |  |  |  |  |
| At week 12 | -404.5 (-679.6 to -129.4) | -37.7 (-328.5 to 253.2) | 366.84 (-33.71 to 767.39) | 0.071 | 0.198 |
| At week 24 | -406.2 (-625.1 to -187.3) | -158.3 (-389.7 to 73.2) | 247.90 (-70.88 to 566.67) | 0.123 |  |
| **IgA change from baseline at week 24, G/L** | -0.37 (-0.63 to -0.12) | -0.58 (0.82 to -0.33) | -0.20 (-0.56 to 0.16) | 0.260 |  |
| **IgM change from baseline at week 24, G/L** | -0.09 (-0.22 to -0.05) | -0.16 (-0.29 to -0.03) | -0.07 (-0.26 to 0.12) | 0.444 |  |
| **IgG change from baseline at week 24, G/L** | -1.59 (-2.77 to -0.41) | -2.01 (-3.16 to -0.85) | -0.42 (-2.08 to 1.25) | 0.616 |  |
| **Anti-CCP antibody change from baseline at week 24, U/ml** | -10.1 (-24.7 to 4.5) | -33.8 (-48.0 to -19.5) | -23.68 (-44.12 to -3.26) | 0.078 |  |

^a^ Data are presented as least squares mean (95% CI) unless stated otherwise.

^b^ For continuous variables, treatment differences across time points were evaluated using a mixed model for repeated-measures analysis. For categorical variables, Generalized Estimation Equations (GEE) was used for analysis.

Abbreviations: ACR, American College of Rheumatology; EULAR, European League Against Rheumatism; ACR20/50/70, ≥20%/50%/70% improvement in the American College of Rheumatology criteria; CCP, cyclic citrullinated peptide; CDAI, Clinical Disease Activity Index; CI, confidence interval; DAS28, Disease Activity Score using 28 joints; ESR, erythrocyte sedimentation rate; GA, global assessment of disease activity; HAQ-DI, Health Assessment Questionnaire-Disability Index; Ld-IL2, low-dose interleukin-2; mITT, modified intent-to-treat; MTX, methotrexate; RF, rheumatoid factor; SDAI, Simplified Disease Activity Index; SF-36 PCS, Short Form-36 physical component scores; SF-36 MCS, Short Form-36 mental component scores; SJC, swollen joint count; TJC, tender joint count; VAS, Visual Analogue Scale.

# Table S4. Factors predicting potential response to low-dose interkleukin-2 treatment in RA.

| **Characteristic** | **Responder**  **n=11** | **Non-responder**  **n=10** | **p-value** |
| --- | --- | --- | --- |
| **Duration, months** | 94 (34, 183) | 63 (47, 317.5) | 0.605 |
| **ESR, mm/H** | 30 (17, 42) | 49 (23.75, 74.5) | 0.114 |
| **CRP, mg/L** | 11.34 (2,14, 31.22) | 18.11 (6.12, 42.11) | 0.557 |
| **DAS28-ESR** | 4.90 (4.78, 5.69) | 5.55 (4.95, 6.50) | 0.132 |
| **RF, IU/ml** | 197.0 (20, 489) | 410.5 (37, 1677.5) | 0.282 |
| **anti-CCP, U/ml** | 211.8 (178.4, 249.4) | 200.7 (145.8, 228.3) | 0.349 |
| **Tregs, % in CD4+ T cells** | 5.83% (4.28%, 6.65%) | 7.46% (5.95%, 8.97%) | **0.036** |
| **IL-2, pg/ml** | 9.66 (5.80, 13.73) | 12.99 (5.60, 29.64) | 0.426 |
| **IL-6, pg/ml** | 45.90 (29.54, 147.49) | 43.32 (23.14, 193.05) | 1.000 |
| **IL-17A, pg/ml** | 84.88 (40.03, 300.20) | 111.17 (8.23, 244.48) | 0.557 |
| **IL-21, pg/ml** | 10.80 (4.10, 22.84) | 3.57 (1.81, 7.15) | **0.012** |
| **TNF-α, pg/ml** | 353.48 (171.02, 591.15) | 379.45 (149.89, 522.33) | 0.654 |
| **IFN-γ, pg/ml** | 11.11 (3.42, 24.35) | 4.78 (1.45, 29.48) | 0.468 |
| **sCD25, ng/ml** | 5.56 (1.94, 12.18) | 2.19 (1.26, 5.13) | 0.314 |
| **IL-7, pg/ml** | 7.15 (4.58, 11.83) | 6.45 (3.77, 9.72) | 0.633 |
| **IL-4, pg/ml** | 43.09 (5.36, 102.38) | 19.44 (6.61, 116.01) | 0.829 |
| **IL-10, pg/ml** | 16.46 (4.54, 42.11) | 13.09 (3.76, 41.26) | 1.000 |
| **IL-12, ng/ml** | 14.18 (7.69, 30.77) | 20.45 (7.57, 58.12) | 0.696 |
| **TGF-β, ng/ml** | 1.76 (0.72, 2.21) | 1.99 (1.63, 2.76) | 0.460 |
| **CXCL13, pg/ml** | 69.03 (38.27, 127.95) | 90.12 (33.06, 110.21) | 0.762 |

Abbreviations: CCP, cyclic citrullinated peptide; CRP, C reactive protein; DAS28, Disease Activity Score using 28 joints; ESR, erythrocyte sedimentation rate; IL, interleukin; Ld-IL2, low-dose interleukin; RA, rheumatoid arthritis; Tregs, regulatory T cells; TGF, transforming growth factor; TNF, tumor necrosis factor.

# Table S5. Change in Cytokines after Low-dose IL-2 Treatment.

| **Characteristics**^a^ | **Baseline** | **Week 12** | **Week 24** | **P value**  **(baseline-week 12)** | **P value**  **(baseline-week24)** | **P value**^b^ |
| --- | --- | --- | --- | --- | --- | --- |
| **IL-2, pg/ml** |  |  |  |  |  |  |
| IL-2+MTX | 10.15 (5.96, 14.85) | 8.56 (6.11, 15.08) | 6.96 (5.75, 10.09) | 0.301 | **0.026** | 0.346 |
| placebo+MTX | 8.86 (4.06, 20.90) | 8.81 (4.17, 15.28) | 7.57 (4.24, 15.68) | 0.614 | 0.627 |  |
| **IL-6, pg/ml** |  |  |  |  |  |  |
| IL-2+MTX | 47.22 (34.07, 200.00) | 73.05 (24.95, 139.43) | 43.91 (26.24, 61.98) | 0.134 | 0.136 | 0.194 |
| placebo+MTX | 61.57 (28.99, 181.66) | 37.26 (13.43, 113.87) | 36.93 (11.53, 151.67) | 0.067 | 0.218 |  |
| **IL-17A, pg/ml** |  |  |  |  |  |  |
| IL-2+MTX | 116.28 (38.28, 274.14) | 112.25 (11.91, 199.59) | 102.15 (20.64, 179.12) | **0.004** | 0.177 | 0.790 |
| placebo+MTX | 143.59 (10.28, 264.05) | 54.13 (7.91, 192.04) | 51.16 (9.24, 168.55) | 0.173 | 0.455 |  |
| **IL-21, pg/ml** |  |  |  |  |  |  |
| IL-2+MTX | 7.39 (4.1, 16.82) | 5.96 (4.56, 21.95) | 6.5 (4.56, 13.03) | 0.737 | 0.363 | 0.233 |
| placebo+MTX | 5.02 (2.10, 10.81) | 6.2 (2.39, 13.78) | 4.79 (2.59, 9.82) | 0.679 | 1.000 |  |
| **TNF-α, pg/ml** |  |  |  |  |  |  |
| IL-2+MTX | 407.29 (237.44, 574.59) | 346.33 (152.10, 517.10) | 324.94 (186.00, 509.56) | **0.020** | 0.093 | 0.579 |
| placebo+MTX | 348.42 (141.87, 597.98) | 269.97 (108.91, 552.78) | 229.76 (86.92, 617.47) | 0.073 | 0.135 |  |
| **IFN-γ, pg/ml** |  |  |  |  |  |  |
| IL-2+MTX | 8.61 (3.27, 23.51) | 6.61 (2.53, 12.52) | 6.54 (2.72, 12.39) | **0.028** | 0.093 | 0.386 |
| placebo+MTX | 5.45 (0.75, 31.02) | 2.20 (0.63, 29.00) | 2.77 (0.69, 15.01) | 0.108 | 0.520 |  |
| **sCD25, ng/ml** |  |  |  |  |  |  |
| IL-2+MTX | 3.76 (2.00, 7.16) | 3.19 (1.26, 5.68) | 3.13 (1.43, 5.10) | 0.050 | 0.177 | 0.407 |
| placebo+MTX | 1.39 (0.66, 8.02) | 1.37 (0.43, 2.62) | 1.52 (0.71, 4.54) | 0.411 | 0.717 |  |
| **Table S5. Continued.** |  |  |  |  |  |  |
| **Characteristics**^a^ | **Baseline** | **Week 12** | **Week 24** | **P value**  **(baseline-week 12)** | **P value**  **(baseline-week24)** | **P value**^b^ |
| **IL-7, pg/ml** |  |  |  |  |  |  |
| IL-2+MTX | 6.81 (4.46, 11.59) | 7.03 (3.20, 10.18) | 6.15 (2.37, 9.98) | 0.509 | 0.522 | 0.642 |
| placebo+MTX | 6.09 (5.16, 7.47) | 5.94 (4.58, 8.43) | 6.62 (4.29, 8.48) | 0.540 | 0.719 |  |
| **IL-4, pg/ml** |  |  |  |  |  |  |
| IL-2+MTX | 26.93 (6.28, 101.91) | 27.80 (6.47, 34.94) | 21.13 (4.32, 42.07) | 0.051 | 0.267 | 0.712 |
| placebo+MTX | 14.18 (3.15, 128.26) | 7.95 (2.51, 113.98) | 6.8 (2.90, 121.00) | 0.064 | 0.716 |  |
| **IL-12, ng/ml** |  |  |  |  |  |  |
| IL-2+MTX | 14.86 (8.67, 50.83) | 5.49 (2.42, 37.25) | 7.03 (1.32, 37.12) | **0.015** | 0.069 | 0.780 |
| placebo+MTX | 12.60 (2.68, 38.73) | 13.62 (1.94, 52.05) | 12.91 (1.62, 35.67) | 0.624 | 0.484 |  |
| **TGF-β, ng/ml** |  |  |  |  |  |  |
| IL-2+MTX | 1.85 (0.92, 2.20) | 1.77 (0.88, 2.42) | 1.75 (0.90, 2.58) | 0.917 | 0.775 | 0.948 |
| placebo+MTX | 1.77 (0.65, 2.55) | 2.38 (1.66, 2.84) | 1.77 (0.58, 2.66) | 0.085 | 0.868 |  |
| **CXCL13, pg/ml** |  |  |  |  |  |  |
| IL-2+MTX | 88.09 (46.14, 98.13) | 52.25 (36.62, 188.10) | 62.43 (45.42, 225.86) | 0.576 | 0.440 | 0.863 |
| placebo+MTX | 69.64 (42.79, 98.86) | 52.09 (35.64, 116.65) | 57.85 (35.08, 81.10) | 0.496 | 0.750 |  |

^a^ Data are presented as median (IQR). ^b^ Treatment differences across time points were evaluated using a mixed model for repeated-measures analysis, with visit, treatment group, treatment-by-visit interactions included in the model.

# Table S6. Inclusion and Exclusion Criteria.

| **Inclusion criteria** |
| --- |
| 1. Male or female ≥18 and ≤70 years of age at time of screening. |
| 2. Diagnosed with rheumatoid arthritis as determined by meeting 1987 American College of Rheumatology (ACR) classification criteria. |
| 3.  Moderate rheumatoid arthritis during screening, as defined by a disease activity score (28 joint) calculated using erythrocyte sedimentation rate formula (DAS28-ESR) > 3.2. |
| 4.  Have given written informed consent. |
| **Exclusion criteria** |
| 1. Patient presenting or having a history of other inflammatory joint disease. |
| 2. Patient with ongoing or previous Stevens-Johnson syndrome, toxic epidermal necrolysis or erythema multiform. |
| 3. Patient with significantly impaired bone marrow function or significant anemia, leucopenia or thrombocytopenia due to causes or other than active rheumatoid arthritis. |
| 4. Persistent infection or severe infection within 3 months before enrollment. |
| 5. Uncontrolled hypertension, uncontrolled diabetes, unstable ischemic heart disease, active inflammatory bowel disease, active peptic ulcer disease, terminal illness or other medical condition which, in the opinion of the investigator, would put the patient at risk to participate in the study. |
| 6. Clinically relevant cardiovascular, hepatic, neurological, endocrine, or other major systemic disease making implementation of the protocol or interpretation of the study results difficult. |
| 7. Severe hypoproteinemia (e.g. in case of severe liver disease or nephrotic syndrome) with serum albumin < 30 g/L. |
| 8. Moderate or severe impairment of renal function, as known by serum creatinine > 133μmol/L (or 1.5 mg/dl). |
| 9. Patient with history of recent and clinically significant drug or alcohol abuse. |
| 10. Impairment of liver function or persisting ALT (SGPT) elevations of more than 2-fold the upper limit of normal. |
| 11. Pregnancy. |
| 12. Breastfeeding. |
| 13. Women of childbearing potential, except if they fulfill specific conditions. |
| 14. Men wishing to father children during the course of the study or within the 24 months thereafter (or 3 months with the washout procedure) |
| 15. Patient with a congenital or acquired severe immuno-deficiency, a history of cancer or lymphoproliferative disease, or any patient who has received total lymphoid irradiation. |
| 16. Known HIV positive status. |
| 17. Known positive serology for hepatitis B or C. |
| 18. Patient with hypersensitivity to any of the excipients in the tablets of methotrexate. |
| 19. Prior treatment with any kind of biologics, such as anti-TNF, abatacept, tocilizumab or rituximab, etc., within 3 months before enrollment. |
| 20. Enrollment in any other clinical trial involving off-label use of an investigational drug or device, or enrollment in any other type of medical research.  21. Any active infection (including chronic or localized infections) for which anti-infectives were indicated within 28 days prior to first investigational product dose. |
| 22. BMI (body mass index) under 18.5 kg/m^2^ or more than 30 kg/m^2^. |

# Table S7. Antibodies Used in Flow Cytometric Analysis.

| **Target antigen** | **Fluorochrome** | **Vendor** |
| --- | --- | --- |
| **assay for patients** |  |  |
| CD3 | Alexa Fluor 700 | Biolegend |
| CD4 | FITC | Biolegend |
| CD8 | PerCP | Biolegend |
| CD25 | PE | Biolegend |
| CD127 | Brilliant Violet 605 | Biolegend |
| CD45RA | Brilliant Violet 510 | Biolegend |
| CXCR3 | PE-CF594 | Biolegend |
| CCR6 | Brilliant Violet 650 | Biolegend |
